# Asymmetric-Fluidic-Reservoirs Induced High Rectification Nanofluidic Diode

**DOI:** 10.1038/s41598-018-32284-7

**Published:** 2018-09-17

**Authors:** Vishal V. R. Nandigana, Kyoo Jo, Aaron Timperman, Narayana R. Aluru

**Affiliations:** 1 Department of Mechanical Engineering, Indian Institute of Technology, Madras, Chennai, 600036 India; 2 U.S. Army Corps of Engineers, Construction Engineering Research Laboratory, 2902 Newmark Drive, Champaign, Illinois 61826 USA; 3 Department of Chemistry, University of Illinois at Urbana-Champaign, Urbana, 6180 USA; 4 Department of Mechanical Science and Engineering, Beckman Institute for Advanced Science and Technology, University of Illinois at Urbana-Champaign, Urbana, 6180 USA

## Abstract

We demonstrate a novel nanofluidic diode that produces rectification factors in excess of 1000. The nanofluidic diode consists of ion permselective nanopores that connect two reservoirs of different diameters- a micropore reservoir and a macropore reservoir. On the application of +100 V to the micropore, a low OFF state current is observed. The OFF state is caused by formation of the ion depleted zone in the micropore because the anions are prevented from entering the nanopores from the micropore and the cations are depleted in this region to maintain charge neutrality. On the application of −100 V, we observe a high ON state current. The ON state is caused by formation of the ion enriched zone in the microchannel because the anions cannot pass through the nanopores and accumulate in the microchannel. To maintain charge neutrality the cations also become enriched in the microchannel. The ratio of ON state current to the OFF state current gives the rectification of current. Here, plasma oxidation is used to achieve a nanopore with a large wall surface charge density of *σ*_*n*_ = −55 *mC*/*m*^2^ which yields a rectification of current on the order of 3500 that is nearly two orders of magnitude higher than those reported thus far. In contrast to the other nanofluidic diodes, this nanofluidic diode does not introduce asymmetry to the nanopore, but asymmetry is produced by having the nanopores join a micropore and a macropore. Introduction of asymmetry into the fluidic reservoirs which the nanopores connect is quite simple. Hence, the nanofluidic diode is easy to scale up to industrial level.

## Introduction

Nanofluidic diodes were invented a decade ago. The early advent of nanofluidic diodes saw the use of conical nanopores^1–5^, conical pipettes^6–9^ and conical nanotubes^10,11^. Conically shaped pores have a diameter of the order of few nanometers on the “tip” side and a diameter of few microns on the “base” side. These devices achieve higher currents when the electric field is directed from tip to base direction. The current is called ON state current. However, the currents reduced when the electric field is from base to tip direction. The current is called OFF state current. The ratio of ON state current to the OFF state current is called current rectification and the rectification of current in conical nanofluidic diodes is because of the conical geometry and is about 100. Another class of nanofluidic diodes includes asymmetric surface charged nanopores^12–16^. In these devices, half of the nanopore wall is coated with biotin, which imparts a neutral surface charge; while the other half of the nanopore is coated with avidin which imparts a positive surface charge. The asymmetric surface charge results in higher current when the voltage is directed from biotin to avidin, while current reduced when the electric field is from avidin to biotin. Enrichment of ions inside the nanopore causes higher current, while depletion of ions inside the nanopore when the polarity of electric field is reversed leads to lower current. The maximum rectification of current in asymmetric surface charged nanofluidic diodes is about 150. The third class of nanofluidic diode is field-effect reconfigurable nanofluidic diode^17–19^. Here, rectification of the current is regulated by the application of gate voltage on top of the nanopore and a maximum rectification of 200 is achieved with these diodes. However, an important limitation of these three classes of nanofluidic diodes is the fabrication because in all these three classes of diodes asymmetry is being introduced to the nanopore. Hence, industrial scale up of these diodes is difficult.

**Figure 1:**
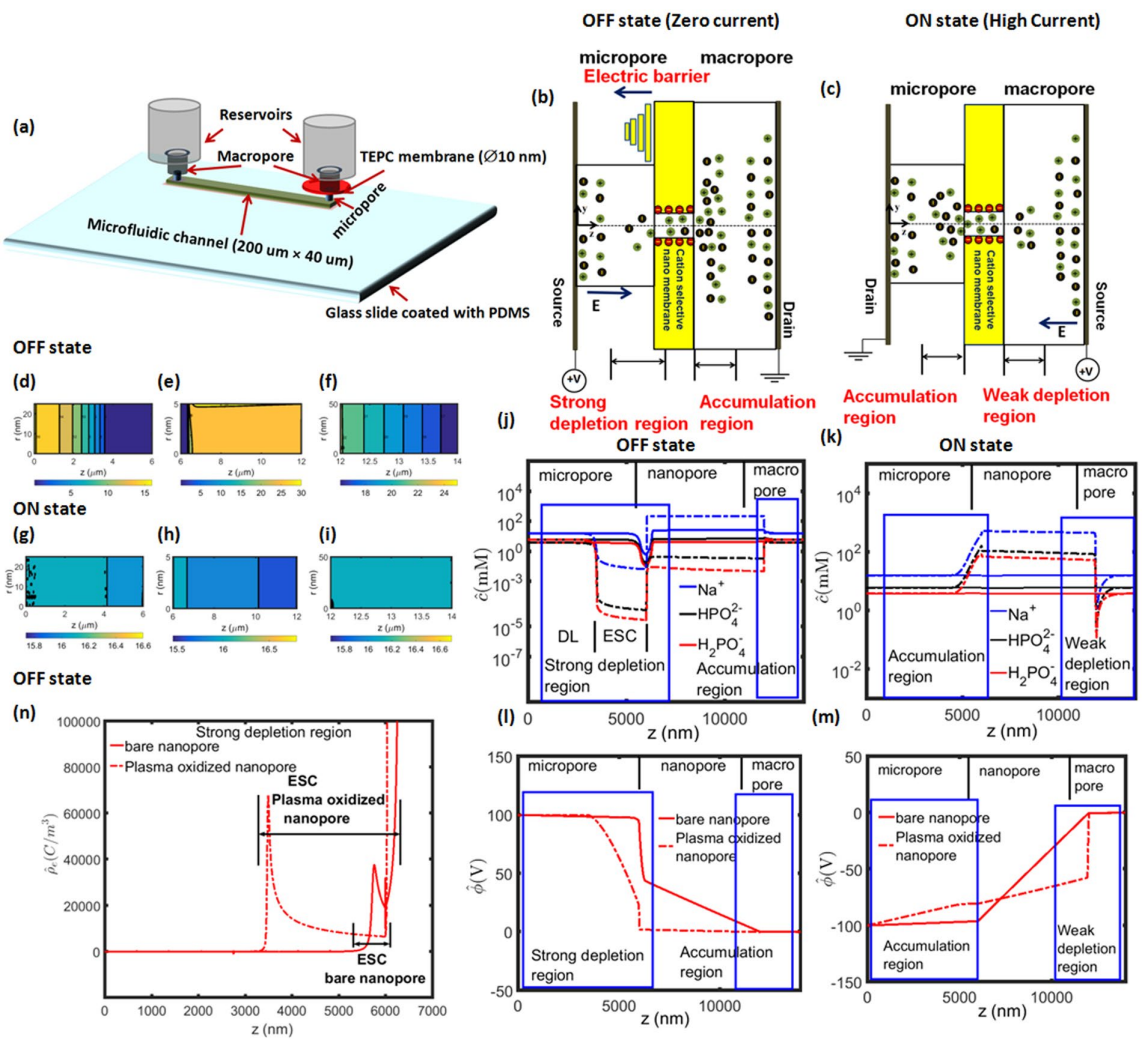
(**a**) 3D schematic representation of experimental nanofluidic diode. Schematic cartoon representation of (**b**) OFF state (**c**) ON state of the nanofluidic diode. The cartoon shows the novel ion concentration polarization (ICP) phenomenon which includes accumulation and depletion region at the micro or macro and nanofluidic interfaces. 2D numerical simulation of OFF state showing (d) depletion region in micropore (**e**) cation-selective nanopore and (**f**) accumulation region in macropore. 2D numerical simulation of ON state showing (**g**) accumulation region in micropore (**h**) cation-selective nanopore and (**i**) depletion region in macropore. Comparison of 1D area-averaged concentration distribution for plasma oxidation nanopore (dashed lines) and bare nanopore (solid lines) in (**j**) OFF state and (**k**) ON state. Comparison of 1D area-averaged potential distribution between plasma oxidation nanopore (dashed lines) and bare nanopore (solid lines) in (**l**) OFF state and (**m**) ON state. (**n**) Numerical simulations of extended space charge region (ESC) for plasma oxidation nanopore (dashed lines) and bare nanopore (solid lines) in OFF state. The simulations are done at *c*_0_ = 10 *mM* solution and at 100 V applied voltage.

Here, we demonstrate a novel nanofluidic diode. Our nanofluidic diode consists of a polycarbonate track etched (TEPC) nanopore membrane that connects two reservoirs of different diameters-a micropore reservoir and a macropore reservoir (see Fig. 1(a)). Our nanofluidic diode is also treated with plasma oxidation. Plasma oxidation results in a variety of functional groups like C-O, C=O, O-C=O, C-O-O that increase the nanopore wettability and zeta potential through the increased number of negatively charged oxygen functional groups. Hence, we produce nanopores with a large wall surface charge density of *σ*_*n*_ = −55 *mC*/*m*^2^. The high nanopore wall surface charge density leads to giant rectification of current of the order of 3500 which is nearly two orders of magnitude higher than current state of the art technology. Also, our nanofluidic diode does not introduce asymmetry in the nanopore unlike the current nanofluidic diodes. Additionally, the nanofluidic diode presented here utilizes asymmetry at the reservoir geometry level rather than within the nanopores, which greatly simplifies fabrication^20,21^. Further, our nanofluidic diode can be scaled up to industrial level. These results show that high rectification factors can be achieved with a simple device design that can be easily multiplexed, overcoming two important hurdles for future translation of these devices into useful system. We envision that these nanopore diodes will be used in new age integrated fluidic circuits and sensor applications.

## Working Principle of The Nanofluidic Diode

The working principle of the nanofluidic diode is based on widely known ion-concentration polarization (ICP)^22–36^. In electrokinetic systems, ICP refers to the depleted and enriched zones that form on opposite sides of an ion permselective material, such as a nanoporous membrane^22–36^. To briefly explain the phenomenon, we take advantage of Fig. 1(b) which gives a schematic cartoon representation of the nanofluidic diode in the OFF state. Here, the electric field is applied from micropore towards macropore. Due to this field, the anions are repelled from the negatively charged nanopore and are attracted towards the anode. This causes a depletion of ions near the anode-nanopore interface. To maintain electro-neutrality the cations also deplete at this interface (see numerical results Fig. 1(d–f)). The electroneutral region is called electroneutral diffusion layer (DL). However, the most crucial part of our observation is that when the nanopore is treated with plasma oxidation the nanopore wall surface charge density is very large of the order of *σ*_*n*_ = −55 *mC*/*m*^2^. As we observe in Fig. 1(j,n), the large nanopore wall surface charge density breaks down the electro-neutral diffusion layer (DL) and forms a concentration polarization based depletion extended space charge region (ESC). The ESC region contains a small amount of net charge which is very important in the diode working principle as it controls the flow of ions, because the ESC region creates a large voltage barrier (see Fig. 1(l)) and resists the ion transport leading to almost zero current in the OFF state.

Now, in the ON state, we switch the polarity of the electric field, i.e., we apply electric field from macropore towards micropore. The region of ESC is reversed and is towards the macro-nanopore interface as shown in schematic Fig. 1(c) and numerical results Fig. 1(g–i). We observe a small depletion extended space charge region in the ON state when compared to the OFF state as shown in Fig. 1(k). A fundamental reason for this observation is because of the larger size of the macropore reservoir compared to the micropore reservoir. The weak depletion extended space charge region results in weak voltage barrier as shown in Fig. 1(m). Hence, we observe weak resistance to the flow of ions and high ON state current. The purpose of the nanofluidic diode is to have high ON state current and almost zero OFF state current. We develop novel theoretical models to calculate the OFF state and ON state currents to achieve high rectification of current.

## Theoretical Model

The OFF state current is given by,

1 $${I}_{ESC}^{OFF}=\frac{5\pi }{1000}(\frac{{\varphi }_{OFF}^{2}{{\rm{\Omega }}}_{Na}+{{\epsilon }}_{0}{{\epsilon }}_{r}}{{L}_{ESC}})\,$$

where *ϕ*_*OFF*_ is the voltage in the OFF state, ${{\rm{\Omega }}}_{N{a}^{+}}$ is the electrical mobility of the sodium ions. We relate the electrical mobility of the sodium ions to the diffusion coefficient $({D}_{N{a}^{+}})$ using Einstein’s relation, ${{\rm{\Omega }}}_{N{a}^{+}}=\frac{e{D}_{N{a}^{+}}}{{k}_{B}T}$, *e* is the electronic charge, *k*_*B*_*T* is the thermal energy, ${{\epsilon }}_{0}$ is the permittivity of free space, ${{\epsilon }}_{r}$ is the permittivity of water, *L*_*ESC*_ is the length of the ESC region. The details of the derivation are given in the supplementary information. The main observation of our theoretical model is that the OFF state current is inversely proportional to the length of the ESC region, *L*_*ESC*_. Hence, we conclude that in order to achieve zero OFF state current *L*_*ESC*_ must be infinite. In our experiments we reach theoretical limit of *L*_*ESC*_ of 70 *mm* which is two orders of magnitude higher than any reported results on ICP so far^34–36^. We also show that *L*_*ESC*_ is related to the nanopore wall surface charge density, *σ*_*n*_. For this we assume that some charge from the nanopore leaks into the micropore to form the ESC region. Hence, we equate the charge in the ESC (*Q*_*ESC*_) as a product of nanopore wall surface charge density times surface area of the nanopore (*d*S) times the leakage factor, *α*;

2 $${Q}_{ESC}=\alpha {\sigma }_{n}dS$$

The charge, (*Q*_*ESC*_) can be theoretically related to the number of sodium ions per unit volume in the ESC $({n}_{ESC}^{N{a}^{+}})$ as,

3 $${Q}_{ESC}={n}_{ESC}^{N{a}^{+}}e\,{A}_{ESC}{L}_{ESC}$$

where *A*_*ESC*_ is the area of the ESC region. This area is also equal to the area of the micropore because the depletion extended space charge region occurs in the micropore. We assume ${n}_{ESC}^{N{a}^{+}}\,$in terms of the bulk concentration $({c}_{0}^{+})$,

4 $${n}_{ESC}^{N{a}^{+}}={c}_{0}^{+}{N}_{A}$$

where *N*_*A*_ is the Avogadro number. Substituting Eq. (4) in Eq. (3) we obtain,

5 $${Q}_{ESC}={c}_{0}^{+}{N}_{A}e{A}_{ESC}{L}_{ESC}$$

From Eqs (2) and (5), we arrive at an expression for length of ESC region, *L*_*ESC*_ in terms of the nanopore wall surface charge density (*σ*_*n*_),

6 $${L}_{ESC}=\frac{\alpha {\sigma }_{n}dS}{{c}_{0}^{+}{N}_{A}e{A}_{ESC}}$$

Eq. (6) shows a linear scaling relation between the length of ESC region, *L*_*ESC*_ and the nanopore wall surface charge density, *σ*_*n*_. We would like to increase the nanopore wall surface charge density so that the length of ESC region, *L*_*ESC*_ is large and the OFF state current is almost zero. In order to achieve this, we treat the nanopore with plasma oxidation. In the present work *L*_*ESC*_ and *σ*_*n*_ are considered as free parameters and are obtained from two sets of experiments, a native nanopore without plasma oxidation and treating the nanopore with plasma oxidation.

We briefly highlight the theoretical model to derive expressions for the ON state current. The complete derivation of ON state current is given in the supplementary information. The main objective of our nanofluidic diode is to maximize the ON state current. We account for the interaction of the ionic concentration (*c*_*i*_) with the applied voltage (*ϕ*_*ON*_) and also the interaction of the nanopore wall surface charge density (*σ*_*n*_) with the applied voltage (*ϕ*_*ON*_). We do not account for the length of the depletion extended space charge region in the macro-nanopore junction as the depletion is weak in the ON state as discussed earlier. Under these assumptions, the ON state current is given by,

7 $${I}_{ON}=\frac{{A}_{n}{F}^{2}{\varphi }_{ON}}{{L}_{n}}[(\sum {z}_{i}^{2}{\mu }_{i}{c}_{i})+(\frac{4{z}_{N{a}^{+}}{\mu }_{N{a}^{+}}|{\sigma }_{n}|}{F{d}_{n}})]$$

*A*_*n*_ is the area of the nanopore, *F* is the Faraday’s constant, *ϕ*_*ON*_ is the voltage in the ON state, *L*_*n*_ is the length of the nanopore, *z*_*i*_ is the valence of the ionic species *i*, *μ*_*i*_ is the mobility of each ionic species given by Einstein’s relation^22^, ${\mu }_{i}=\frac{{D}_{i}}{RT}$, *D*_*i*_ is the diffusion coefficient of each ion, *R* is the gas constant, *T* is the temperature, *c*_*i*_ is the concentration of each ionic species, ${z}_{N{a}^{+}}$ is the valence of the sodium ion, ${\mu }_{N{a}^{+}}$ is the mobility of the sodium ion $({\mu }_{N{a}^{+}}=\frac{{D}_{N{a}^{+}}}{RT})$, ${D}_{N{a}^{+}}$ is the diffusion coefficient of sodium ion, *d*_*n*_ is the diameter of the nanopore. Eq. (7) shows that the ON state current is directly proportional to the nanopore wall surface charge density. Thus, in order to maximize ON state current we must have large nanopore wall surface charge density which is achieved by our plasma oxidation method.

## Results and Discussion

### Native/Bare Nanopores (or Low Surface Charge Nanopores)

**Figure 2:**
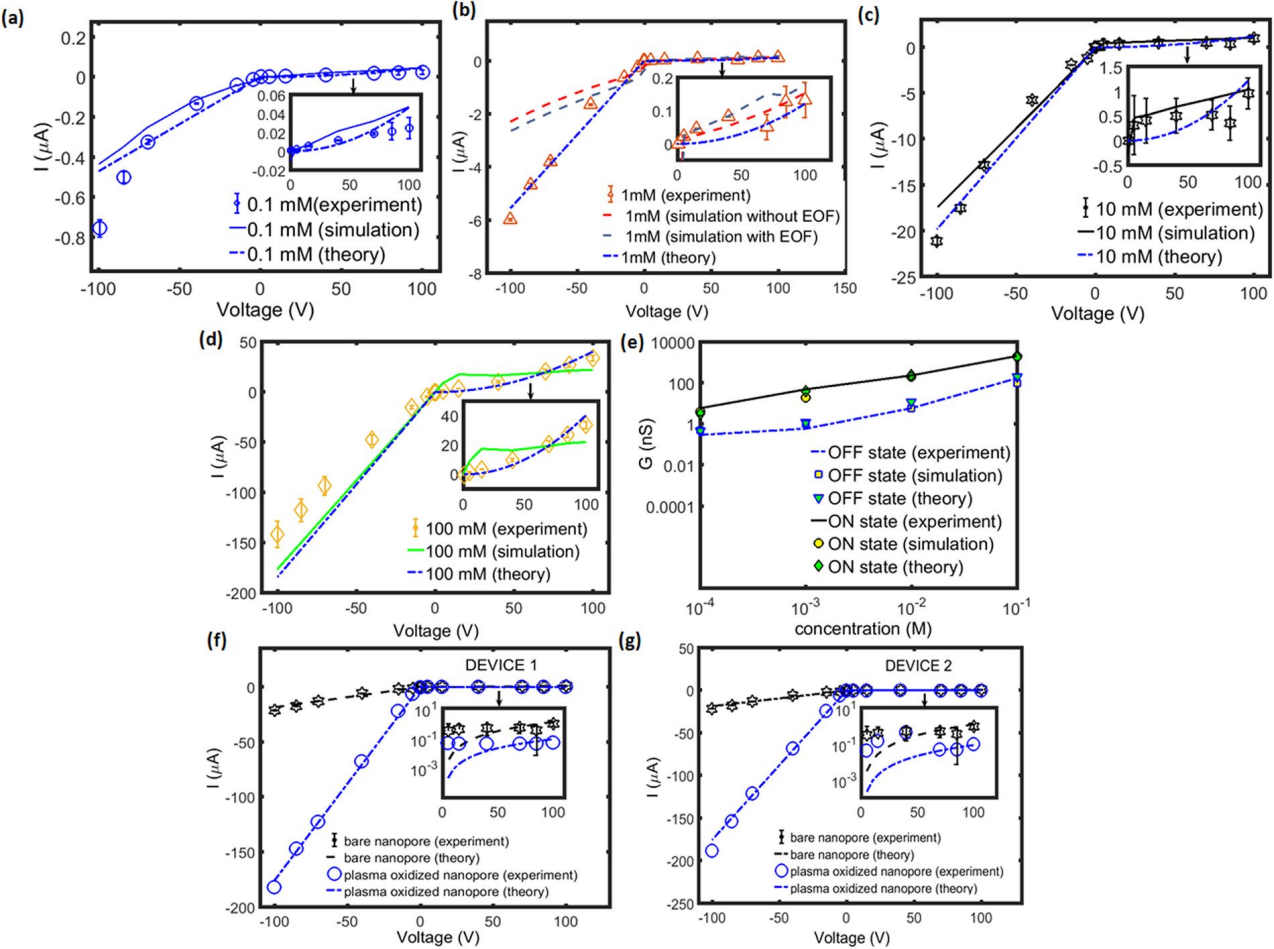
ON/OFF state current-voltage characteristics of bare nanopore under different concentration, (**a**) 0.1 *mM* (**b**) 1 *mM* (**c**) 10 *mM* (**d**) 100 *mM*. All the current-voltage curves are validated with numerical simulations (solid lines) and theory (dashed lines). We include an additional effect of electroosmotic flow (EOF) in our numerical simulation at 1 mM concentration to match the experimental results. The EOF effect is included in the numerical simulation because the nanopore wall surface charge density is highest at this concentration as shown in Table 1. (**e**) Relationship of conductance with concentration for ON and OFF state, respectively. (**f**,**g**) Comparison of current-voltage characteristics for plasma oxidized nanopore with bare nanopore for two separate devices. The ON state current of plasma oxidized nanopore in both the devices is one order of magnitude higher than bare nanopore. Similarly the OFF state current is one order of magnitude lower than bare nanopore. The giant ON state current and the low OFF state current are observed due to the giant nanopore wall surface charge density obtained by plasma oxidation method. The concentration for this case is 10 *mM*.

We perform our first set of experiments with native nanopores, i.e., without plasma oxidation to gain insights into the current-voltage characteristics. Figure 2(a–d) shows the current-voltage characteristics for different concentrations ranging from 0.1 *mM* to 100 *mM*. We observe that OFF state current is small (not zero) for all the concentrations. We fit the two free parameters, length of ESC region, *L*_*ESC*_ and nanopore wall surface charge density, *σ*_*n*_ from our experimental data to gain knowledge about these parameters. The values of the two data are tabulated in Table 1. We observe a maximum surface charge density of *σ*_*n*_ = −1.3 *mC*/*m*^2^ at 1 *mM* concentration. The nanopore wall surface charge density is not large enough to observe zero OFF state current because at the given nanopore wall surface charge density, the electroneutral diffusion layer does not break down into large ESC region which is evident from the length of the depletion extended space charge region tabulated in Table 1. Further, we observe a decrease in length of ESC region with concentration because the bulk conduction is larger at higher concentration which leads to stronger electroneutral diffusion layer.

**Table 1:** Numerical values of the two free parameters, length of ESC region, *L*_*ESC*_ and nanopore wall surface charge density, *σ*_*n*_ obtained from experiments.

Concentration (*mM*)	Surface charge density (*mC/m*^2^)	Length of ESC region (*mm*)	Surface charge density (*mC/m*^2^)	Length of ESC region (*mm*)
Bare nanopore			Plasma oxidized nanopore	
0.1	−0.1	13		
1	−1.3	5		
10	−0.5	0.5	−55	70
100	−0.05	0.015		

Now, let us look at the ON state, we observe relatively high ON state current when compared to the OFF state current owing to the contribution of nanopore wall surface charge density and the second term in Eq. (7). However, the current is still not higher to observe a giant ON state current as the nanopore wall surface charge density is very small. Further, Fig. 2(e) shows the ON state conductance scaling with concentration. We observe a linear scaling of ON state current with the concentration at high concentration limit beyond 1 *mM* and a saturation of current is observed at low concentration limit, (below 1 *mM*) The observations are consistent with other solid-state nanopores^36^ primarily due to the fact that the bulk concentration term dominates at high concentration limit, the first term in Eq. (7) and nanopore wall surface charge density dominates at low concentration limit, the second term in the Eq. (7). Further, Table 1 shows that the nanopore wall surface charge density has a non-monotonic trend with concentration because the zeta potential (*ζ*) decreases with increase in concentration, an observation postulated by Keesom *et al*.^37^ on polycarbonate track etched membranes. The nanopore wall surface charge density is related to the zeta potential using Grahame equation^38^,

8 $$|{\sigma }_{n}|={[2{{\epsilon }}_{0}{{\epsilon }}_{r}{k}_{B}T\sum _{i=1}^{n}{n}_{{o}_{i}}(\exp (-\frac{{{\rm{z}}}_{{\rm{i}}}e{\rm{\zeta }}}{{{\rm{k}}}_{{\rm{B}}}{\rm{T}}})-1)]}^{\frac{1}{2}}$$

${n}_{{o}_{i}}$ is the number density of the ionic species, *i*, *n* is the number of ionic species.

We also carry out numerical simulations based on Poisson-Nernst-Planck equations in OpenFOAM^24–26^ to validate our experiments and theoretical models. Figure 2(a–e) shows the numerical results agreeing well with the theory and experiments. However, we observe deviation between the numerical simulations and experiments for 1 mM concentration (see Fig. 2(b)) due to the fact that we had considered a simple Helmholtz-Smoluchowski electroosmotic flow equation given by Eq. 14 to model the fluidic flow. These deviations should be addressed with a more accurate model which is part of our future work.

### Plasma oxidized nanopores (or High Surface Charge Nanopores)

Our next set of experiments involves plasma oxidation treatment of nanopores. Figure 2(f) shows the current-voltage characteristics for the plasma oxidized nanopore in comparison to the native nanopore. We observe an order of magnitude increase in the ON state current and an order of magnitude reduction in the OFF state current when compared to the bare nanopore. We fit the OFF and ON state current value to Eqs (1) and (7), respectively to obtain the two free parameters, length of ESC region, *L*_*ESC*_ and nanopore wall surface charge density, *σ*_*n*_. The results are tabulated in Table 1. We observe that the theoretical value of *L*_*ESC*_ is 70 *mm* which is almost two orders of magnitude higher than bare nanopore and *σ*_*n*_ is determined to be *σ*_*n*_ = −55 *mC*/*m*^2^, which is also two orders of magnitude higher than bare nanopores. The large ESC region and the giant nanopore wall surface charge density are due to our novel plasma oxidation treatment of the nanopore. These two parameters result in almost zero OFF state current of 51.8 *nA* and a giant ON state current of 181.8 *μA*. The net result is a high rectification of current of about 3500 which is two orders of magnitude higher than those reported thus far. These results demonstrate that high surface charge is critical for achieving high rectification factors with asymmetric concentration polarization. With the present device design, we did observe the ON/OFF state currents decrease as a function of time and improving the stability of these devices will be a goal of future work.

**Figure 3:**
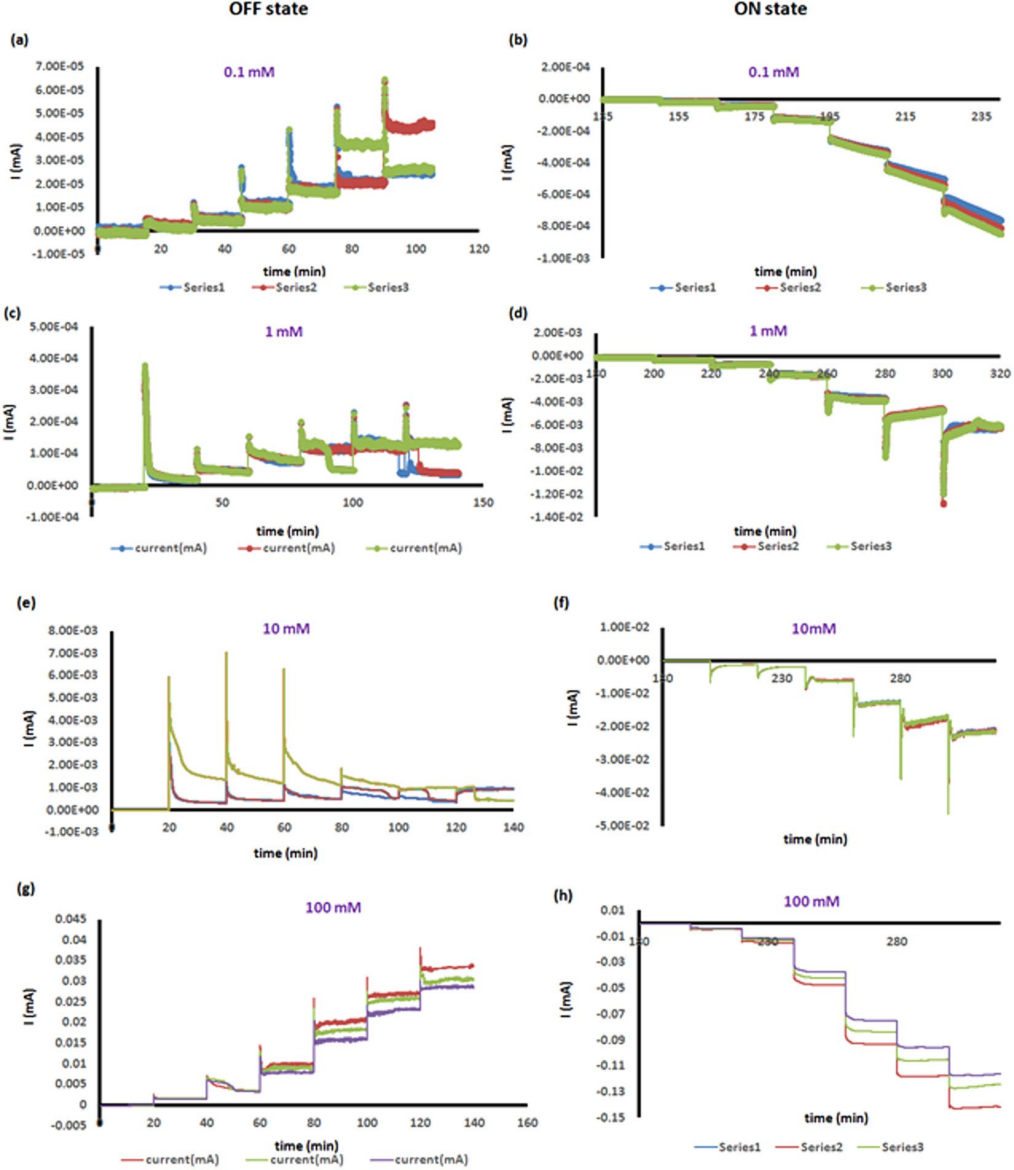
(**a**–**h**) OFF/ON state current-time response of bare nanopores without plasma oxidation. We collect the current-time response for 3 trials at each voltage in either OFF/ON state. A voltage is applied for 10 minutes for the current to reach equilibrium. A voltage step of 0.5 V, 5 V, 15 V, 40 V, 70 V, 85 V and 100 V are applied one by one in both OFF and ON state, respectively.

**Figure 4:**
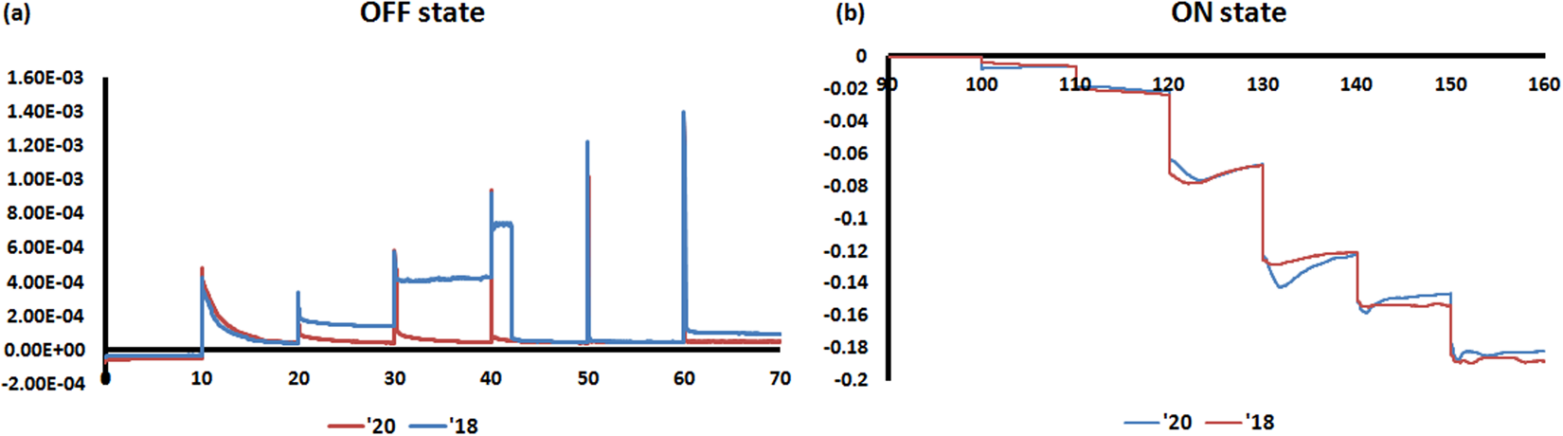
(**a**,**b**) OFF/ON state current-time response of the plasma oxidized nanopores. We collect the current-time response at each voltage for 2 different devices (device ‘20 and device ‘18). A voltage is applied for 10 minutes for the current to reach equilibrium. The voltage step is same as Fig. 3.

Figures 3 and 4 shows the current-time plots indicating the nanofluidic diode response as a function of time for both bare and plasma oxidized nanopores, respectively. Previously, we have shown that response time is a function of device design and that the response decreases as the rigidity of the nanopore membrane increases^39^. Here we find the response time is also dependent on the concentration and that the response time decreases as the concentration of the phosphate buffer increases from 0.1 to 100 mM range. At 500 mM the ICP is insufficient and the response time is not considered because the current rectification falls below 10. At 100 mM the response time is less than one second and future studies with data acquisition rates greater than the 1 Hz used here will be required to more precisely determine the response time.

## Conclusion

We demonstrate a novel nanofluidic diode that can achieve rectification factors in excess of 1000. Our nanofluidic diode consists of a nanopore connected to two reservoirs of different diameters- a micropore reservoir and a macropore reservoir. Further, we treat the nanopore by plasma oxidation to achieve a large nanopore wall surface charge density of *σ*_*n*_ = −55 *mC*/*m*^2^. The high nanopore wall surface charge density gives for the first time a giant ON state current and almost zero OFF state current. Also, our nanofluidic diode does not introduce asymmetry to the nanopore unlike the current nanofluidic diodes. Our nanofluidic diode introduces asymmetry at the reservoir geometry level which is easy to fabricate and scale up to industrial level.

## Methods

### Materials

Fluorescein sodium salt is purchased from Sigma-Aldrich (Milwaukee, WI). The phosphate buffer had a pH of 7.0 and 0.1–100 mM concentrations are used unless otherwise stated. Agarose gel is purchased from Sigma-Aldirch (Milwaukee, WI). Tri-decafluoro-1,1,2,2-tetrahydroctyl-1-trichlorosilane is purchased from United Chemical Technologies (Bristol, PA). 18 MΩcm distilled deionized water is produced with a Millipore Milli-Q^®^ direct water purification system (Billerica, MA). SU-8 2050 photoresist and SU-8 developer are purchased from MicroChem Corp. (Westborough, MA). The track-etched polycarbonate (TEPC) nanocapillary membrane (NCM) with 10 nm ID pores are purchased from GE water & Process Technologies Inc. (Trevose, PA). The 4-inch silicon wafer were obtained from Addison Engineering (San Jose, CA).

### Device fabrication

The devices were fabricated using standard photolithography and PDMS casting methods as previously reported^40^. Briefly, the masters for casting the PDMS are fabricated by creating features on the silicon substrate with the SU-8 2050 photoresist. The 3-D photoresist masters required 2 layers of photoresist. In the first layer, the SU-8 that forms the mold for the microchannel (which is an extension of the micropore) is deposited and developed. In the second layer circular SU-8 features are deposited on top of the previous SU-8 layer at each end of the microchannel and development.

A thin PDMS layer that forms the horizontal microfluidic channel and the macropore is cast on the SU-8 on silicon masters using a 10:1 mixture of PDMS prepolymer and curing agent. This thin PDMS layer is formed by spin-coating the SU-8 master with uncured PDMS mixture for 30 seconds at 1100 rpm, followed by curing at 95 °C on a hotplate for 5 minutes. The cured 65 *μm* thick PDMS layer is carefully removed from the master wafer in isopropanol solvent and transferred onto a Corning glass slide (Corning, NY) pre-coated with a thin PDMS layer. Thus, the thin PDMS layer is attached to the PDMS layer on top of the glass and all surfaces of the microfluidic channel are PDMS. The substrate with the NCM attached is plasma-treated (using a PDC-32 G plasma cleaner, Harrick Scientific, Ossining, NY) and a track-etched polycarbonate NCM is centered on top of the vertical microfluidic channel (which is referred to as the micropore in this article). The top substrate PDMS layer is approximately 3 mm and has the reservior and macropore holes punched in it. All layers of the device and the NCM, which is attached to the thin middle layer, are treated for 10 s in the plasma cleaner and are pressed together within less than 1 minute after plasma treatment for permanent bonding. The horizontal microchannel is 200 *μm* wide, 40 *μm* in depth, and 2 cm in length and is terminated with a verical microfluidic channel (macropore) that has a 150 *μm* ID and 25 *μm* height. The nanopores in the TEPC-NCM have a 65 *nm* ID, 6 *μm* length, and pore density of 6 × 10^8^ *pores*/*cm*^2^ as reported by the manufacturer.

To make the current measurements,the fluidic system was filled with the pH 7.2 phosphate buffer. The data with the plasma oxidized NCMs was acquired with a buffer of 10 mM. Additional studies, were performed using buffer concentrations of 0.1 mM, 1 mM, and 100 mM as noted. Pt wire electrodes were inserted into the inlet reservoir and the macropore reservior. The voltage steps were applied with an in-house program written in LabWindows CVI (National Instruments). The voltage program steps were 0, 5, 15, 40, 70, 85, 100, −100 (clean), 0 (diffuse), 0, −5, −15, −40, −70,− 85, −100, 100 (clean), 0 (diffuse), and successive data sets were collected automatically in an interative manner. The high voltage was applied with reference to the inlet resevoir while the macropore electrode was always grounded. Thus the OFF state currents were acquired with negative applied voltage and the ON state current were applied with positve applied voltage. The step length varied from 10–20 minutes as indicated in the current-time (i-t) plots while the last 60 points of each voltage step was used to calculate the average current in the current-voltage (i-v) plots. The clean and diffuse steps were added to reduce the hysteris and start each deviation from 0 volts with a similar initial state. The voltage was applied with a Trek Model 220 high voltage amplifier, and the current was recorded with an Agilent 34410A digital multimeter at a frquency of 1 hz.

### Numerical model

We develop a novel ion transport model^22–26^ to predict the ON/OFF state currents. In this model, we neglect the contribution of *H*^+^ and *OH*^−^ ions as its concentration is much lower compared to the bulk concentration of the ionic species. Hence, the water dissociation effects are not considered in the numerical model. Further, we assume that the ions are immobile inside the Steric layer and do not contribute to the ionic current. We also do not model the Faradic reactions occurring near the electrode. Finally, we assume that the electroosmotic component of current originating from fluid flow is only present at 1 mM concentration as this is the concentration where we observe a maximum nanopore wall surface charge density. Under these assumptions, the total flux of each ionic species (**Γ**_*i*_) is contributed by a diffusive component resulting from the concentration gradient and an electrophoretic component arising due to the potential gradient as given by Eq. (9)

9 $$\,{{\rm{\Gamma }}}_{i}=-\,{D}_{i}\frac{\partial \widehat{{c}_{i}}}{\partial z\,}+{{\rm{\mu }}}_{i}{z}_{i}F\widehat{{c}_{i}}\hat{E}$$

where $\hat{f}=\frac{1}{{\rm{\Delta }}({\rm{z}})}{\int }_{0}^{R}{\int }_{0}^{2\pi }f(r,\theta )rdrd\theta$ is the area averaged quantity. ${\hat{c}}_{i}$ is the area-averaged concentration of the *i*^*th*^ species, $\hat{E}$ is the area-averaged electric field and Δ(z) is the cross sectional area accounting for the nanopore and the reservoir geometries. In order to find the concentration we equate the mass transport of the ionic species with the flux,

10 $${\rm{\Delta }}(z)\frac{\partial \widehat{{c}_{i}}}{\partial t}=\frac{\partial }{\partial z}({\rm{\Delta }}(z){D}_{i}\frac{\partial \widehat{{c}_{i}}}{\partial z})-\frac{\partial }{\partial z}({\rm{\Delta }}(z){z}_{i}F\widehat{{c}_{i}}{{\rm{\Omega }}}_{{\rm{i}}}\hat{E})$$

Ω_*i*_ is the ionic mobility which is related to the Einstein’s equation by ${{\rm{\Omega }}}_{i}=\frac{{D}_{i}}{RT}$. Equation (10) is the area-averaged Nernst-Planck equation. Elaborate details regarding the derivation of area-averaged equations are discussed in our earlier works^25,26^. The individual ionic current (***I***_*i*_) across the reservoir and the nanopore is calculated by integrating their respective fluxes over the respective cross−sectional area, i.e.

11 $${{\boldsymbol{I}}}_{i}=\int {z}_{i}F{{\boldsymbol{\Gamma }}}_{i}dS$$

The total ionic current at any axial location is calculated from individual ionic currents, ${I}_{tot}={\sum }_{i=1}^{n}{I}_{i}$. In order to determine the area-averaged electric field in Eq. (9), we solve the area-averaged Poisson equation,

12 $$\frac{\partial }{\partial z}({\rm{\Delta }}(z){{\epsilon }}_{r}\hat{E})=\frac{{\rm{\Delta }}(z)}{{{\epsilon }}_{0}}(\widehat{{\rho }_{e}}+\frac{2{\sigma }_{s}(z)}{R(z)})$$

where $\widehat{{\rho }_{e}}$ is the net space charge density of the ions defined as, $\widehat{{\rho }_{e}}={\sum }_{i=1}^{n}{z}_{i}F\widehat{{c}_{i}}$ and *R*(*z*) is the radius of the micro, nano or macropore, respectively. The second term on the right hand side in Eq. (12) is obtained after invoking the Gauss law on the membrane surface, ${{\epsilon }}_{r}{\rm{E}}\cdot \overrightarrow{n}=\frac{-{\sigma }_{s}(z)}{{{\epsilon }}_{0}}$, where *σ*_*s*_(*z*) is the surface charge density of micro, nano or macropore surface. $\overrightarrow{n}$ is the unit normal in the *r* direction. We couple the area-averaged Nernst-Planck equation, Eq. (10) with the area-averaged Poisson equation, Eq. (12) to obtain the area-averaged ionic concentration, electric potential and ionic currents. Now to account for the electroosmotic flow, we bridge the ionic current and the electroosmotic axial flow velocity, *u*_*z*_ using an area-averaged Navier-Stokes equation. We neglect the pressure gradient and assume a fully developed flow in the axial direction. This gives the 1-D Navier-Stokes equation as,

13 $$\mu (\frac{1}{r}\frac{{\rm{\partial }}}{{\rm{\partial }}r}(r\frac{{\rm{\partial }}{u}_{z}}{{\rm{\partial }}r}\,))={\epsilon }_{r}{\epsilon }_{0}(\frac{1}{r}\frac{{\rm{\partial }}}{{\rm{\partial }}r}(r\frac{{\rm{\partial }}\varphi }{{\rm{\partial }}r}\,)){{\rm{E}}}_{{\rm{z}}}$$

where *μ* is the viscosity, *ϕ* is the electrical potential. The equation is formulated by assuming that the electric field *E*_*z*_ varies only in the axial mode, and the potential *ϕ* constitutes the Debye layer potential. By this we mean, the charges in the perpendicular direction to the applied electric field are constituted only near the nanopore surface giving rise to a potential near the surface, and zero potential away from the surface. The distance until which the potential exists is given by the Debye length, ${\lambda }_{D}={(\frac{{{\epsilon }}_{o}{{\epsilon }}_{r}RT}{{\sum }_{i=1}^{n}{F}^{2}{z}_{i}^{2}{c}_{0}})}^{1/2}$, where *c*_0_ is the bulk concentration. This electric potential acting along with the axial electric field, *E*_*z*_ constitutes the body force on the fluid. The other assumptions for the fluid flow are the flow is steady and incompressible. The boundary conditions are given by; at the centre of the membrane domain, $r=0;\frac{\partial \varphi }{\partial r}=0$ (since the potential is axisymmetric) and $\frac{\partial {u}_{z}}{\partial r}=0$ (as the fluid flow is axisymmetric). The other boundary condition is at the shear plane; *r* = *R*; *ϕ* = *ζ* and *u*_*z*_ = 0. *u*_*z*_ = 0 implies that there is no slip velocity at the shear plane. This reduces the electroosmotic flow on the pore surface to Helmholtz Smoluchowski equation^41^;

14 $${u}_{z}=\frac{-{{\epsilon }}_{0}{{\epsilon }}_{r}\zeta {E}_{z}}{\mu }$$

We assume the same electroosmotic flow throughout the entire radial direction. Next, we couple the electroosmotic flow velocity, *u*_*z*_ with the area-averaged Nenrst-Planck equation, Eq. (10) by introducing a convective flow term; *c*_*i*_*u*_*z*_. We substitute the Helmholtz Smoluchowski equation for *u*_*z*_. We arrive at a newly modified-Nernst-Planck equation (MNP) including the electroosmotic flow.

15 $${\rm{\Delta }}(z)\frac{\partial \widehat{{c}_{i}}}{\partial t}=\frac{\partial }{\partial z}({\rm{\Delta }}(z){D}_{i}\frac{\partial \widehat{{c}_{i}}}{\partial z})-\frac{\partial }{\partial z}({\rm{\Delta }}(z){z}_{i}F\widehat{{c}_{i}}{{\rm{\Omega }}}_{{\rm{i}}}\hat{E})+\frac{\partial }{\partial z}({\rm{\Delta }}(z)\widehat{{c}_{i}}\frac{{{\epsilon }}_{0}{{\epsilon }}_{r}\zeta \widehat{{E}_{z}}}{\mu })\,$$

Eq. (15) is coupled with the area-averaged Poisson equation, Eq. (12) to calculate the ionic current with electroosmotic flow. Finally, we provide boundary conditions for the closure of the problem. The bulk concentration at the ends of the micro and macro reservoir is given by; *c*_*bulk*_. We give a voltage *ϕ*_*opplied*_, at the end of the micropore and the macropore is grounded. This represents the OFF state. We apply a negative potential, −*ϕ*_*opplied*_ at the end of the micropore and keep the macropore grounded. This represents the ON state. Thus, the boundary conditions at the ends of the micro (Eq. (16)) and macro (Eq. (17)) reservoirs are specified as:

16 $$\,{c}_{i}={c}_{bulk};\varphi =0$$

17 $${c}_{i}={c}_{bulk};\varphi =\pm \,{\varphi }_{applied}$$

The coupled MPNP equations are numerically coded in OpenFOAM^42^ (OpenField Operation and Manipulation) as our earlier works^25,26^.

### Simulation parameters

Sodium phosphate buffer solution is used in all the simulations. The bulk concentration of the micro/macro reservoir is varied from 0.1 *mM* to 100 *mM*. The simulation temperature is *T* = 300 *K*. The bulk diffusivities of *Na*^+^, ${H}_{2}P{O}_{4}^{-}$ and $HP{O}_{4}^{2-}$ are $1.33\times {10}^{-9}\,{m}^{2}/s$, $\,0.879\times {10}^{-9}{m}^{2}/s$ and $0.439\times {10}^{-9}{m}^{2}/s$, respectively. The dielectric constant of the aqueous solution is ${{\epsilon }}_{r}=80$. The simulated domain consists of a nanopore of length *L*_*n*_ = 6 *μm* and diameter *d*_*n*_ = 10 *nm* The diameter of the micropore is *d*_*micro*_ = 10 *nm*. The length of the micropore is *L*_*micro*_ = 6 *μm*. The diameter of the macropore is *d*_*macro*_ = 100 *nm*. The length of the macropore is *L*_*macro*_ = 2 *μm*. We observe that the diameter of the micro/macropore is of the order of few hundreds of nanometers since we calculate the ionic current across a single nanopore that is exposed to the micro/macropore reservoir. However, the experiments are carried out with 106,029 nanopores that are open to the micro/macropore reservoir diameter. We scale up the numerical current with the number of nanopores to match the current for all 106,029 nanopores. The free parameter, nanopore wall surface charge density varied according to the inlet concentration. The nanopore wall surface charge density for each concentration is: ${\sigma }_{n}=-\,1\,mC/{m}^{2}$for 0.1 mM, ${\sigma }_{n}=-\,3\,mC/{m}^{2}$ for 1 mM, ${\sigma }_{n}=-\,2\,mC/{m}^{2}$(OFF) and ${\sigma }_{n}=0\,mC/{m}^{2}$ (ON) for 10 mM, ${\sigma }_{n}=-\,6.38\,mC/{m}^{2}$ (OFF) and ${\sigma }_{n}=0\,mC/{m}^{2}$ (ON) for 100 mM.

## Electronic supplementary material

Supplementary information

Movie of ion-concentration polarization (ICP) in the nanofluidic diode during OFF state
