# Supplementary material for: Asymmetric-Fluidic-Reservoirs Induced High Rectification Nanofluidic Diode
Source: Sci Rep. 2018 Sep 17;8:13941. doi: 10.1038/s41598-018-32284-7 (PMC6141591; doi:10.1038/s41598-018-32284-7)

**Supplimentary information**

Asymmetric-Fluidic-Reservoirs Induced High Rectification

Nanofluidic Diode

Vishal V. R. Nandigana^1*^, Kyoo Jo^2^, Aaron Timperman^3^, Narayana R. Aluru^4^

^1^Department of Mechanical Engineering,

Indian Institute of Technology, Madras, Chennai 600036

^2^U.S. Army Corps of Engineers, Construction Engineering Research Laboratory,

2902 Newmark Drive, Champaign, Illinois 61826, USA

^3^Department of Chemistry,

University of Illinois at Urbana-Champaign, Urbana 6180, USA

^4^Department of Mechanical Science and Engineering,

Beckman Institute for Advanced Science and Technology,

University of Illinois at Urbana-Champaign, Urbana 6180, USA

**Correspondence should be addressed to* [*nandiga@iitm.ac.in*](mailto:nandiga@iitm.ac.in)*, Ph: +91-44-2257-4668*

**Derivation of OFF state current**

We consider the following assumptions to derive the current in the OFF state. The depletion extended space charge (ESC) region occurs only in the micropore as we assume some of the charge inside the nanopore leaks into the micropore forming the ESC region. Also, we assume the ESC region consists of predominantly cations because the nanopore is permselective to cations. Under these assumptions, we have, $n^{Na^{+}}\left. \right|_{L_{ESC}}=constant$, $n^{ph^{-}}\left. \right|_{L_{ESC}}=0$, where $n^{Na^{+}}$and $n^{ph^{-}}$ are the number of sodium (cations) or phosphate ions (anions) per unit volume, respectively. $L_{ESC}$ is the length of the depletion extended space charge region. The remaining length of the micropore is going to be electro-neutral diffusion layer, hereafter as neutral region, NR, consisting of both cations and anions and its length is given as; $L_{NR}$. The total length of the micropore is therefore, $L_{mi}=L_{ESC}+L_{NR}$. We further assume that only diffusion governs the transport in the neutral region and there is no electric field $(E)$ drop in this region; i.e., $E=0$. As a result for a positive bias, $\phi_{applied}$ in the micropore, the boundary condition is; $\phi\left. \right|_{x=0}=\phi\left. \right|_{x=L_{NR}}=0$ and $\left. \phi\right|_{x=L_{NR}}=\phi_{applied}$. Based on this assumption, the current in the OFF state in the ESC region is expressed in terms of total ionic charge ${(z_{Na^{+}}en_{ESC}^{Na^{+}}A}_{ESC}L_{ESC})$ times velocity $(v_{ESC})$ normalized by length of ESC region $(L_{ESC})$. $z_{Na^{+}}$ is the valence of the sodium ion, $e$ is the electronic charge, $n_{ESC}^{Na^{+}}$ is the total number of sodium ions in the ESC per unit volume, $A_{ESC}$ is the area of the ESC, also the area of the micropore as the ESC region occurs in the micropore. The velocity $(v_{ESC})$ is assumed to be equal to the electrical mobility of the ion, $\Omega_{Na^{+}}\left( =\frac{eD_{Na^{+}}}{k_{B}T} \right)$ times the electric field,$E_{ESC}$. $v_{ESC}=\Omega_{Na^{+}}E_{ESC}$^S1^. $D_{Na^{+}}$ is the diffusion coefficient of sodium ion, $k_{B}T$ is the thermal energy. Therefore, the current in the OFF state is given by,

$I_{ESC}^{OFF}=\frac{{z_{Na^{+}}en_{ESC}^{Na^{+}}A}_{ESC}L_{ESC}\Omega_{Na^{+}}E_{ESC}}{L_{ESC}}$ (S1)

We require $n_{ESC}^{Na^{+}}$ and electric field $E_{ESC}$ in Eq. (S1) to calculate the OFF state current. $E_{ESC}$ can be obtained by solving the Poisson equation in the axial direction$(z)$,

$\frac{d^{2}\phi}{dz^{2}}=-\frac{z_{Na^{+}}en_{ESC}^{Na^{+}}}{\epsilon_{0}\epsilon_{r}}$ (S2)

where, $\epsilon_{0}$ is the permittivity of the free space and $\epsilon_{r}$ is the permittivity of the medium (water). Integrating the above equation along $z$ direction gives,

$E\left( z \right)=\frac{z_{Na^{+}}en_{ESC}^{Na^{+}}z}{\epsilon_{0}\epsilon_{r}}$ (S3)

Since, $\frac{\partial\phi}{\partial z}=-E$. Substituting the electric field $E$ from Eq. (S3) in Eq. (S1) results,

$I_{ESC}^{OFF}=\frac{{z_{Na^{+}}^{2}e}^{2}\left( n_{ESC}^{Na^{+}} \right)^{2}A_{ESC}\Omega_{Na^{+}} z}{\epsilon_{0}\epsilon_{r}}$ (S4)

Integrating Eq. (S4) along z direction from $z_{NR}$to $z_{ESC}$ on both sides, and substituting the distance between $z_{NR}$ to $z_{ESC}$ as the length of ESC, $L_{ESC}$ gives,

$I_{ESC}^{OFF}=\frac{{z_{Na^{+}}^{2} e}^{2}\left( n_{ESC}^{Na^{+}} \right)^{2}A_{ESC}\Omega_{Na^{+}}L_{ESC}}{2\epsilon_{0}\epsilon_{r}}$ (S5)

We do not know the value of, $n_{ESC}^{Na^{+}}$, the total number of sodium ions in the ESC per unit volume to calculate the OFF state current. In order to find this value we consider the nanopore region. The total charge $\left( Q_{total} \right)$ stored inside the nanopore is given by,

$Q_{total}=|\sigma_{n}|dS=z_{Na^{+}}en_{nano}^{Na^{+}}dV_{nano}$, (S6)

where $\sigma_{n}$ is the nanopore wall surface charge density, $dS$ is the nanopore surface area. The total charge $Q_{total}$ is also equal to $z_{Na^{+}}en_{nano}^{Na^{+}}dV_{nano}$, where $n_{nano}^{Na^{+}}$the number of sodium ions per unit volume in the nanopore and ${dV}_{nano}$ is the volume of the nanopore. We assume that the total charge, leaks into the ESC region with a leakage factor $\alpha$, given by,

$\alpha=\frac{Q_{ESC}}{Q_{total}}$ (S7)

where $Q_{ESC}$is the total charge in the ESC region which is given as,

$Q_{ESC}= z_{Na^{+}}en_{ESC}^{Na^{+}}A_{ESC}L_{ESC}$ (S8)

We substitute Eq. (S7) in Eq. (S8) which gives,

$n_{ESC}^{Na^{+}}=\frac{\alpha Q_{total}}{z_{Na^{+}}eA_{ESC}L_{ESC}}$ (S9)

Substituting for $n_{ESC}^{Na^{+}}$ in Eq. (S5), we obtain the current in the OFF state,

$I_{SCR}^{OFF}=\frac{\alpha^{2}\left( Q_{total} \right)^{2}\Omega_{Na^{+}}}{2\epsilon_{0}\epsilon_{r}A_{ESC}L_{ESC}}$ (S10)

We have all the inputs to calculate the current in the OFF state, except the leakage factor, $\alpha.$We continue our analysis to obtain the constraints on $\alpha$. The two constraints on $\alpha$are :

$\alpha>0$ (S11)

$\alpha<\frac{Q_{ESC}^{max}}{Q_{total}}$, (S12)

The first constraint is that $\alpha$ is positive. The second constraint shows the maximum ESC, $Q_{ESC}^{max}$ that can be leaked inside the micropore. We have,

$Q_{ESC}^{max}=|\sigma_{ESC}^{max}|dS_{ESC}$ (S13) where$\sigma_{ESC}^{max}$ is the maximum surface charge density in the ESC region and $dS_{ESC}$ is the surface area of the ESC. To obtain $\sigma_{ESC}^{max}$, we invoke the Gauss law which is given by,

$\epsilon_{r}E_{\mathrm{ESC}}^{max}\cdot\vec{n}=\frac{{-\sigma}_{ESC}^{max}}{\epsilon_{0}}$ (S14)

where $E_{\mathrm{ESC}}^{max}$ is the maximum electric field in ESC and $\vec{n}$ is the unit vector in the normal direction. Substituting Eq. (S14) in Eq. (S13) we get,

$Q_{ESC}^{max}= \epsilon_{r}\epsilon_{0}E_{\mathrm{ESC}}^{max}dS_{ESC}$ (S15)

Now, if we assume all electric field drops across the ESC, $E_{ESC}^{max}=\frac{\phi_{applied}}{L_{ESC}}$, then there is no electric field drop in the nanopore, $E_{nano}=0$, which is not physical. Hence, $E_{ESC}^{max}<\frac{\phi_{applied}}{L_{ESC}}$. Substituting for maximum electric field drop condition in the ESC region, $E_{ESC}^{max}$in Eq. (S15), we obtain,

$Q_{ESC}^{max}<\frac{\epsilon_{r}\epsilon_{0}\phi_{applied}dS_{ESC}}{L_{ESC}}$ (S16)

We substitute Eq. (S16) in Eq. (S12) to obtain an upper limit for $\alpha$;

$\alpha<\left( \frac{{\epsilon_{0}\epsilon_{r}\phi}_{applied}dS_{ESC}}{L_{ESC}Q_{total}} \right)$. Finally, we assume 5% voltage drop in the micropore during the formation of ESC. So, the upper limit of $\alpha$ is given by,

$\alpha= \left( \frac{{{0.05 \epsilon}_{0}\epsilon_{r}\phi}_{applied}dS_{ESC}}{L_{ESC}Q_{total}} \right)$ (S17)

Substituting Eq. (S17) in Eq. (S10), we obtain the OFF state current.

$I_{ESC}^{OFF}=\frac{{0.0025\epsilon_{r}\epsilon_{0}\phi}_{applied}^{2}dS_{ESC}^{2}\Omega_{Na^{+}}}{2A_{ESC}L_{ESC}^{3}}$ (S18)

We substitute $dS_{ESC}=2\pi R_{ESC}L_{ESC}$, where $R_{ESC}$ is the radius of the ESC region and for the cross-sectional area of the ESC region, we substitute $A_{ESC}=\pi R_{ESC}^{2}$. Eq. (S18) reduces to,

$I_{ESC}^{OFF}=\frac{5\pi}{1000}\left( \frac{{\epsilon_{r}\epsilon_{0}\phi}_{applied}^{2}\Omega_{Na^{+}}}{L_{ESC}} \right)$ (S19)

Eq. (S19) shows that the OFF state current is inversely proportional to the length of the ESC region, $L_{ESC}$. Hence, to obtain almost zero OFF state current, $L_{ESC}$ should be very large which is obtained by our novel plasma oxidation method.

**Derivation of ON state current**

We propose a generalized oscillator model to derive the ON state current. The oscillator model is called “Variational non-equilibrium potential oscillator", VN oscillator. The model states that the ionic velocity,$\frac{\partial\vec{x_{i}}}{\partial t}$ along with the total charge of the ionic species, $ez_{i} n_{i}^{total}$ divided by the total length of the system, $L_{total}$ gives the ON state current,

$\vec{I}=\sum_{i=1}^{n} \frac{ez_{i}n_{i}^{total}}{L_{total}}\frac{\partial\vec{x_{i}}}{\partial t}$ (S20)

where, $z_{i}$ is the valence of the ionic species, $i$, $n_{i}^{total}$ is the total number of ions of each ionic species, $i$ and $n$ is the total number of species. We predict the velocity, $\frac{\partial\vec{x_{i}}}{\partial t}$ from the displacement of ion, $x_{i}$. To calculate the displacement, we solve the Newton's equation of motion,

$m_{i}\frac{\partial\vec{x_{i}^{2}}}{\partial t}+ \dot{\lambda}\frac{\partial\vec{x_{i}}}{\partial t}=F_{i}\left. \right|_{t_{n}}$ (S21)

where $m_{i}$is the mass of the each ionic species, $i$, $\dot{\lambda}$, is the damping constant given by, $\dot{\lambda}$ = $\frac{k_{B}T}{D_{i}}$, $D_{i}$ is the diffusion coefficient of each ionic species. $F_{i}\left. \right|_{t_{n}}$is the conservative force acting on the ionic species at time $t_{n}$. $F_{i}\left. \right|_{t_{n}}$is given by the interaction of the ionic charge, $ez_{i}$ with the applied voltage $\left( V_{0} \right)$ plus the interaction of the charge with the nanopore wall surface charge density plus the interaction of the nanopore wall surface charge density with the applied voltage.

$F_{i}\left. \right|_{t_{n}}=\frac{ez_{i}V_{0}}{\left| \vec{x_{b2}}-\vec{x_{b1}} \right|}+\frac{ez_{i}Q_{total}}{4\pi\epsilon_{0}\epsilon_{r}n_{T}^{total}\left| \vec{x_{i}}-\vec{x_{o}^{n}} \right|^{2}}+\frac{Q_{total}V_{0}}{n_{T}^{total}\left| \vec{x_{b2}}-\vec{x_{b1}} \right|}$ (S22)

We apply an electrostatic potential, $V_{0}$ across the micro-nano-macroporous system within a length of $\left| \vec{x_{b2}}-\vec{x_{b1}} \right|$. The variable $\vec{x_{0}^{n}}$ is the position of the nanopore. In 1-D this is equal to; $\vec{x_{0}^{n}} =L_{mi}+L_{n}$, where $L_{mi}$ is the length of the micropore and $L_{n}$ is the length of the nanopore. Also, $\left| \vec{x_{b2}}-\vec{x_{b1}} \right|=L_{mi}+L_{n}+L_{ma}$, where $L_{ma}$ is the length of the macropore. We normalize $Q_{total}$ with the total number of ionic species, $n_{T}^{total}=\sum_{i=1}^{n} c_{o_{i}}\times N_{A}\left[ dV_{mi}+dV_{n}+dV_{ma} \right]$ since we are interested in the force acting on a single ion in Eq. (S22). $c_{0_{i}}$ is the bulk concentration of ionic species, $dV_{mi}$ is the volume of the micropore, $dV_{n}$ and $dV_{ma}$ are the volumes of the nanopore and macropore, respectively and $N_{A}$ is the Avogadro number. The model is marched in time by $\Delta t$ amount using a well know velocity-verlet algorithm^S2^. The position at $t+\Delta t$ is given by,

$\vec{x_{i}}\left( t+\Delta t \right)= \vec{x_{i}(t)}+\vec{\dot{x_{i}}}\left( t \right)\Delta t+0.5\vec{\ddot{x_{i}}}\left( t \right)\Delta t^{2}$ (S23)

The force acting on the ion at time $\left( t+\Delta t \right)$ is calculated using Eq. (S22) at $\vec{x_{i}}\left( t+\Delta t \right)$. Finally, the velocity is given by,

$\vec{\dot{x_{i}}}\left( t+\Delta t \right)= \vec{\dot{x_{i}}}\left( t \right)+0.5\left( \vec{\ddot{x_{i}}}\left( t \right)+\vec{\ddot{x_{i}}}\left( t+\Delta t \right) \right)\Delta t$ (S24)

We considered the following terms to non-dimensionalize the VN oscillator model: distance $x$ is normalized by $x_{\Delta}=1 nm$; time $t$ by $t_{\Delta}=1 ns$; diffusion coefficient$D_{i}$ by $D_{\Delta}=1 nm^{2}/ns$; charge $Q_{total}$ by $q_{\Delta}=\sigma_{n}dS$; and potential $V_{0}$ by applied potential, $V_{0}$. The non-dimensional equation gives us a unique non-dimensional number called Nandigana number, $N=\frac{\sigma_{n}dS}{4\pi\epsilon_{0}\epsilon_{r}x_{\Delta}V_{0}}$. The non dimensional number shows the effect of the nanopore wall surface charge density and the applied electric potential on the ON state current. In the model, we neglect the micropore and the macropore system as they are assumed not to influence the transport in the ON state because the ESC region is weak in the macropore. Also, we reduce the equation to a simple single sodium ion oscillator model instead of the generalized oscillator model as it is much easier to understand. The dynamics of single sodium ion inside the nanopore is given by,

$m_{Na}\frac{\partial\vec{x_{Na}^{2}}}{\partial t}+ \dot{\lambda}_{Na^{+}}\frac{\partial\vec{x_{Na}}}{\partial t}=F_{Na}\left. \right|_{t_{n}}$, (S25)

where $m_{Na}$ is the mass of the sodium ion, $x_{Na}$ is the displacement of the sodium ion, $\dot{\lambda}_{Na^{+}}$ is the damping constant which is given by $\dot{\lambda}_{Na^{+}}$ = $\frac{k_{B}T}{D_{Na^{+}}}$. $F_{Na}\left. \right|_{t_{n}}$is the conservative force acting on the sodium ion at time $t_{n}$, which is given by,

$F_{Na}\left. \right|_{t_{n}}=\frac{ez_{Na^{+}}V_{0}}{\left| \vec{x_{b2}}-\vec{x_{b1}} \right|}+\frac{ez_{Na^{+}}Q_{total}}{4\pi\epsilon_{0}\epsilon_{r}n_{Na}^{total}\left| \vec{x_{Na}}-\vec{x_{o}^{n}} \right|^{2}}+\frac{Q_{total}V_{0}}{n_{Na}^{total}\left| \vec{x_{b2}}-\vec{x_{b1}} \right|},$ (S26)

where $\left| \vec{x_{b2}}-\vec{x_{b1}} \right|$ is the length of the nanopore, $L_{n}$. $\vec{x_{o}^{n}}$ is also the length of the nanopore, $L_{nano}$ and $n_{Na}^{total}=c_{0}^{Na^{+}}\times N_{A}dV_{n}$, where $c_{0}^{Na^{+}}$is the bulk concentration of the sodium ion. The first term shows the interaction of the charged sodium ion with the applied voltage, $V_{0}$. The order of this force is $\left( O({10}^{-13}) \right).$The second term shows the electrostatic interaction of the sodium ion with the nanopore wall surface charge, $Q_{total}$. For a nanopore surface charge density of $\sigma_{s}^{n}= -1.3 mC/m^{2}$, the order of this force is $\left( O({10}^{-19}) \right)$. The third term shows the interaction of the nanopore wall surface charge with the applied voltage. The order of this force is $\left( O({10}^{-13}) \right).$ We observe that the second term is very small compared to the other two terms and hence is neglected in the model.

The ON state current is given by the charge of the sodium ion times the velocity, $\frac{\partial\vec{x_{Na}}}{\partial t}$ divided by the length of the nanopore,

$\vec{I}=\frac{n_{Na}^{total}ez_{Na^{+}}}{L_{nano}}\frac{\partial\vec{x_{Na}}}{\partial t}$ (S27)

Neglecting the acceleration of the sodium ion $\left( \frac{\partial\vec{x_{Na}^{2}}}{\partial t}=0 \right)$ in Eq. (S25) and substituting for $\frac{\partial\vec{x_{Na}}}{\partial t}$ from Eq. (S26) into Eq. (S25), the 1-D ionic current is given by,

$I^{ON} =\frac{n_{Na}^{total}ez_{Na^{+}}}{L_{n}}\left( \frac{ez_{Na^{+}}V_{0}}{\dot{\lambda}_{Na^{+}}L_{n}}+\frac{Q_{total}V_{0}}{n_{Na}^{total}\dot{\lambda}_{Na^{+}}L_{n}} \right)$ (S28)

Substituting Eq. (S6) and $dV_{n}=A_{n}L_{n}$ where $A_{n}$ is the area of the nanopore, Eq. (S28) reduces to,

$I^{ON}=\frac{A_{n}F^{2}V_{0}}{L_{n}}\left[ \left( z_{Na^{+}}^{2}c_{0}^{Na^{+}}\mu_{Na^{+}} \right)+\left( \frac{{4z_{Na^{+}}\mu}_{Na^{+}}\left| \sigma_{s}^{n} \right|}{Fd_{n}} \right) \right]$ (S29)
where $F$ is the Faraday’s constant given by,

$F=eN_{A}$ (S30)

Also, $\mu_{Na}^{+}=\frac{1}{\dot{\lambda}_{Na^{+}}}=\frac{D_{Na^{+}}}{RT}$ is the mobility of the sodium ion and $R$ is the gas constant. Eq. (S29) is very similar to Eq. (7) in the main article, with the only difference Eq. (S29) considers only one ionic species, sodium ion instead of all the ionic species in Eq. (7) in the main article.

**Relationship between nanopore surface charge density and zeta potential: Grahame equation**

The space charge density $(\rho_{e})$ of the ions in the system is given by,

$\rho_{e}= \sum n_{i}z_{i}e$ (S31)

where $n_{i}$ is the concentration of the $i^{th}$ species. Using Boltzmann equation, we approximate the concentration of the each ionic species, $i$, as

$n_{i}=n_{o_{i}}exp\left( -\frac{z_{i}e\psi}{k_{B}T} \right)$ (S32)

where, $n_{o_{i}}$ is the bulk concentration of the ions, $\psi$ is the potential. Using Poisson equation across the plane of the system,

$\frac{d^{2}\psi}{dy^{2}}=-\frac{\rho_{e}}{\epsilon_{0}\epsilon_{r}}$ (S33)

Substituting Eq. (S31) and Eq. (S32) into Eq. (S33),

$\frac{d^{2}\psi}{dy^{2}}= -\frac{1}{\epsilon_{0}\epsilon_{r}}\left( \sum{n_{o}}_{i}z_{i}e exp\left( -\frac{z_{i}e\psi}{k_{B}T} \right) \right)$ (S34)

Now, using the relation, $\frac{d^{2}\psi}{dy^{2}}=\frac{1}{2}\frac{d}{dy}\left( \frac{d\psi}{dy} \right)^{2}$ and substituting in Eq. (S34),

$\frac{1}{2}\frac{d}{dy}\left( \frac{d\psi}{dy} \right)^{2}= -\frac{1}{\epsilon_{r}\epsilon_{0}}\sum n_{o_{i}}z_{i}e exp\left( -\frac{z_{i}e\psi}{k_{B}T} \right)$ (S35)

$\left( \frac{d\psi}{dy} \right)^{2}= \int-\frac{2}{\epsilon_{r}\epsilon_{0}}\sum{n_{o}}_{i}z_{i}e exp\left( -\frac{z_{i}e\psi}{k_{B}T} \right) dy$ (S36)

$\left( \frac{d\psi}{dy} \right)^{2}=\frac{2k_{B}T}{\epsilon_{0}\epsilon_{r}}\sum{n_{o}}_{i}\exp\left( -\frac{z_{i}e\psi}{k_{B}T} \right)+C$ (S37)

Applying the boundary conditions, $\frac{d\psi}{dy}=0$for $y=0$ due to the symmetry of the plane. Substituting the boundary condition in Eq. (S37), we obtain $C$. Eq. (S37) reduces to,

$\left( \frac{d\psi}{dy} \right)^{2}=\frac{2k_{B}T}{\epsilon_{r}\epsilon_{0}}\sum{n_{o}}_{i}\left[ \exp\left( -\frac{z_{i}e\psi}{k_{B}T} \right)-1 \right]$ (S38)

$\frac{d\psi}{dy}$ on the wall can be equated to the wall surface charge density $(\sigma_{n})$ using Gauss law^S1^.

$\frac{d\psi}{dy}\cdot n=\frac{\sigma_{n}}{\epsilon_{0}\epsilon_{r}}$ (S39)

where $n$ denotes unit normal denoting outwards to the planar surface. Subsituting Eq. (S39) in Eq. (S38), we arrive at the Grahame equation^S3^.

$\sigma_{n}= \pm\left[ {2k}_{B}T\epsilon_{0}\epsilon_{r}\sum n_{o_{i}}\left( \exp\left( -\frac{z_{i}e\psi}{k_{B}T} \right)-1 \right) \right]^{\frac{1}{2}}$ (S40)

Eq. (S40) is similar to Eq. (8) in the main article, except that potential $\psi$ is replaced by the zeta potential, $\zeta$.

**Nonlinear electroosmotic flow**

We perform additional 2D simulations to understand nonlinear electroosmotic flow or electroosmotic flow of second-kind. The electroosmotic flow of second kind is a well known phenomenon in concentration polarization dominated system as it arises due to the presence of ESC region^S4,S5^. When the ESC region is acted upon by a tangential electric field we generate a nonlinear fluid flow which is different from the primary electroosmotic flow (EOF). This is because the primary EOF is linearly proportional to the tangential electric field, $E$ while the EOF of second kind scales quadratically with the electric field. The interaction of the nonlinear electroosmotic fluid velocity with the electric field results in a vortex formation. In this study, we use numerical simulations to understand if there is any vortex formation in the nanofluidic diode. Fig. S1 (a) and S1 (b) shows the velocity profile in the micro/nanopore region. We observe uniform flow in the micro/macro as well as nanopore region. Also, we do not observe the electroosmotic flow of second-kind in the nanofluidic diode because there is no vortex formation at the interfaces of the micro-nanopore. Hence, our nanofluidic diode offers tremendous fluidic stability.

***
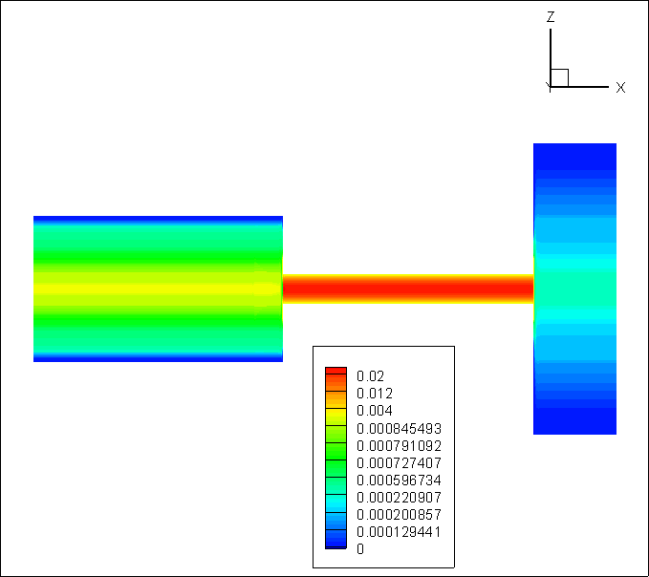

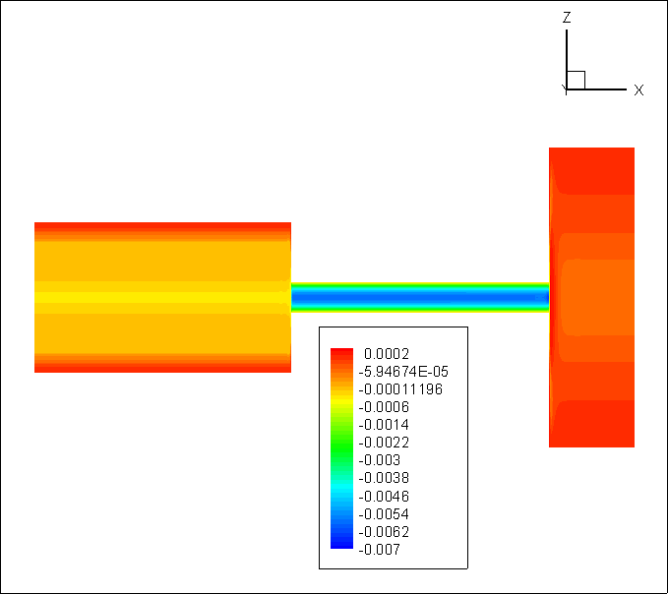
***

**(b)**

**(a)**

Figure S1. 2D velocity profile in (a) OFF state condition and (b) ON state condition. The simulation is done at $1 mM$ sodium phosphate concentration. The label indicates velocity values in $m/s$. We do not observe electroosmotic flow of second-kind in the nanofluidic diode.

**Effect of Agarose gel and PEG on ON/OFF state current**

We carry out additional experiments to understand the effect of highly viscous materials like agarose gel or Polyethylene glycol (PEG) on the ON/OFF state current. Fig. S2 shows the current-voltage characteristics for $10 mM$ solution with and without $1 \%$ agarose gel or $50 \%$ PEG in the macropore. In all the three cases we observe the same OFF state current with no change in the length of ESC region. We postulate the reason to the low Reynolds number as it leads to small convection and rapid stabilization of the ESC region. However, in the ON state, Fig. S2 shows significant reduction in the ON state current owing to the increase in the lengths of the ESC region in the macropore. The primary reason for such increase in lengths of ESC region is the addition of viscous material like agarose gel and PEG suppresses the fluidic convection and increases in the macropore. To gain better insight into these results, Eq. (7) in the main article is used to calculate the ON state current. We used reduced diffusion coefficient for each ion to consider the viscous effects. The individual diffusion coefficient of each ion is multiplied by a factor of 0.016 for agarose gel case and multiplied by a factor of 0.01 for PEG. The OFF state currents were calculated using Eq. (1) in the main article using the same parameters as with the buffer solution case. Fig S2 shows a good match between the theoretical model and the experiments in both ON and OFF state. To our knowledge, these are the first experiments reported for the nanofluidic diode.


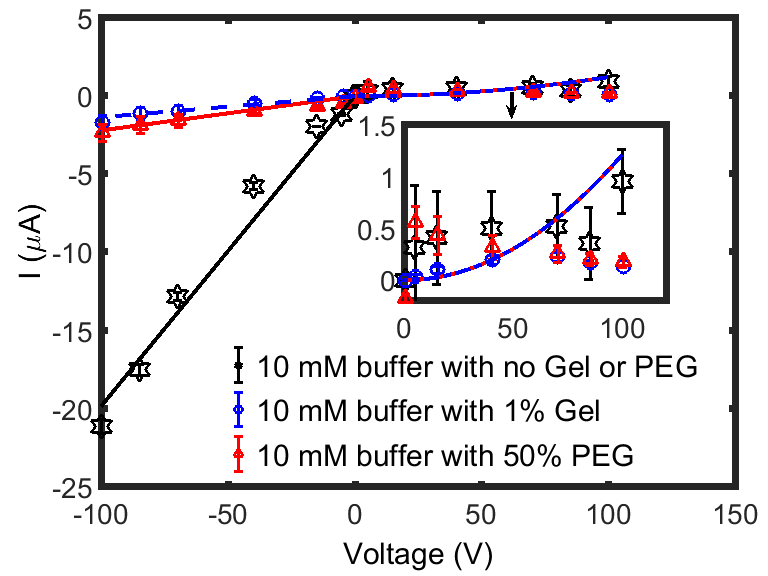


Figure S2. Current-voltage characteristics with 1% agarose gel or 50% PEG along with $10 mM$ buffer solution.

**Joule heating effect**

We also perform additional numerical simulations to understand if there is any Joule heating effect inside the nanofluidic diode to understand the thermal stability of the nanofluidic diode. In our experiments, the temperature of the nanofluidic diode did not rise beyond 10% of the room temperature ensuring the nanofluidic diode is thermally stable. In order to understand numerically the Joule heating effects, we develop an area-averaged Joule heating model. Neglecting the radial convection, the fluid temperature due to convective fluid flow and conductive water is given as;

$\rho c_{p}\left( \frac{dT}{dt}+\boldsymbol{u}\cdot\nabla T \right)=\nabla\cdot\left[ k_{1}\left( T \right)\nabla T \right]+\lambda_{1}E^{2}$ (S41)

where $\rho$ is the density of the fluid, $c_{p}$ is the specific heat of the fluid. $\boldsymbol{u}$ is the velocity vector, $k_{1}$is the thermal conductivity and $\lambda_{1}$ is the electrical conductivity. A linear model is employed to model the temperature dependence on electrical conductivity$(\lambda_{1})$.

$\lambda_{1}=\lambda_{0}(1+\beta\left( T-T_{0} \right))$ (S42)

where $\lambda_{0}$ is the bulk electrical conductivity given by,

$\lambda_{0}=\frac{F^{2}}{RT}\sum_{i=1}^{n} z_{i}c_{i}D_{i}$ (S43)

$c_{i}$ is the concentration of the ionic species and $\beta$ is the temperature coefficient. We assume the velocity of the fluid does not vary across the radial direction, and is given by the Helmholtz-Smoluchowski equation^S6^,

$u_{z}= -\frac{\epsilon_{0}\epsilon_{r}\zeta E_{z}}{\mu}$ (S44)

The boundary conditions at the ends of the micro and macro reservoirs are specified as;

$T_{mi}=T_{0}$ (S45)

$T_{ma}=T_{0}$ (S46)

where $T_{mi}$ is the temperature of the micropore reservoir and $T_{ma}$ is the temperature of the macropore reservoir, $T_{0}=300 K$ is the bulk temperature. The other boundary conditions are,

$\frac{\partial T}{\partial r}\left( 0,z \right)=0$ (axisymmetric boundary condition) (S47)

$\frac{\partial T}{\partial r}\left( R_{n},z \right)= -\frac{h_{1}}{k_{1}}\left( T\left( R_{n},z \right)-T_{0} \right)$ (S48)

$\frac{\partial T}{\partial r}\left( R_{mi/ma},z \right)= 0$ (S49)

Where $R_{n}$ is the radius of the nanopore and $R_{mi/ma}$ is the radius of the micropore or macropore respectively. $h_{1}$ is the heat transfer coefficient of the fluid. We neglect the term related to viscous dissipation (i.e., the thermal energy converted from the mechanical energy) as it is small compared to the Joule heating effect^S7,S8^.

We integrate Eq. (S41) along the $r$ direction to average out the radial effects.

$\int_{0}^{R} \int_{0}^{2\pi} \frac{\partial T}{\partial t}rdrd\theta+ \int_{0}^{R} \int_{0}^{2\pi} u_{z}\frac{\partial T}{\partial z}r dr d\theta=\alpha_{1}\int_{0}^{R} \int_{0}^{2\pi} \frac{\partial^{2}T}{\partial z^{2}}r dr d\theta+ \int_{0}^{R} \int_{0}^{2\pi} \frac{1}{r}\left( \frac{\partial}{\partial r}\left( \alpha_{1}r\frac{\partial T}{\partial r} \right) \right) r dr d\theta+\lambda_{1}\alpha_{1}\int_{0}^{R} \int_{0}^{2\pi} \frac{E^{2}}{k_{1}}rdrd\theta$ (S50)

where $\alpha_{1}=\frac{k_{1}}{\rho c_{p}}$. Substituting the boundary conditions Eq. (S45) to Eq. (S49) and also substituting Eq. (S44) in Eq. (S50), we arrive at an area-averaged Joule heating model,

$\Delta\left( z \right)\frac{\partial\hat{T}}{\partial t}=\frac{\epsilon_{r}\epsilon_{o}\zeta\hat{E}}{\mu}\Delta\left( z \right)\frac{\partial\hat{T}}{\partial z}-2\pi R\left( z \right)\alpha_{1}\left( \frac{h_{1}}{k_{1}}\left( T\left( z \right)-T_{0} \right) \right)+ \Delta\left( z \right)\alpha_{1}\frac{\partial^{2}\hat{T}}{\partial z^{2}}+\frac{\hat{E}^{2}}{k_{1}}\Delta\left( z \right)\alpha_{1}\lambda_{0}(1+\beta\left( T-T_{0} \right))$ (S51)

where Δ(z) is the cross sectional area accounting for the micro, nano and macropore respectively. $R\left( z \right)$ is the radius of the micro-nano-macropore, respectively and $\hat{f}=\frac{1}{\Delta(z)}\int_{0}^{R} \int_{0}^{2\pi} f\left( r,\theta\right)rdrd\theta$ is the area averaged quantity. We consider two approaches to predict the Joule heating effects. One, we consider coupling Eq. (S51) with Eq. (10) and Eq. (12) in the main article to determine the local concentration, electric potential and the electrical conductivity, $\lambda_{0}$. The coupling is a one way coupling, where we assume the fluid properties like diffusivity, thermal conductivity and viscosity are independent of temperature. The second approach is a more realistic approach where we consider a two ways coupling in which the area-averaged Joule heating model is coupled with Eq. (10) and Eq. (12) in the main article along with accounting for the temperature variations of the fluid properties.

The effect of viscosity variation with temperature is modeled using Eq. (S52)^S7^.

$\mu\left( T \right)=2.716\times{10}^{-6}exp\left( \frac{1713}{T} \right)$ (S52)

Similarly, the temperature variation of permittivity $\epsilon(T)$^S8^ and the diffusion coefficient of ionic species $D_{i}(T)$ are given by,

$\epsilon\left( T \right)=305.7 exp\left( -\frac{T}{219} \right)$ (S53)

$D_{i}\left( T \right)={D_{0}}_{i}(1+0.0209\left( T-T_{0} \right))$ (S54)

where, ${D_{0}}_{i}$ is the bulk diffusion coefficient of ionic species $i$ at temperature $T_{0}.$The thermal conductivity, $k_{1}\left( T \right)$, density, $\rho(T)$ as a function of temperature are obtained from the standard steam tables ^S9^. The values for the other simulation parameters are $\zeta= -6.21 mV$^S3^, nanopore wall surface charge density is $\sigma_{n}=0 mC/m^{2}$, bulk concentration is $c_{0}=10 mM$ and input voltage is $\phi=40V$. The heat transfer coefficient at $T_{0}=300 K$ is $h =0.21 W/m^{2}K$; the thermal conductivity is $k_{1}=0.6 W/mK$. $\rho=1000 kg/m^{3}$, $\mu=1.002\times{10}^{-3} Pa\cdot s$and temperature coefficient, $\beta=0.027 K^{-1}$for $10 mM$ concentration^S7^. Fig. S3 (a) shows that the one way coupling approach leads to unphysical results as the temperature rise inside the nanopore is $700 K$. However, the two ways coupling approach in which we account for the temperature variations of the fluid properties yields physically consistent results and the temperature rise inside the nanopore is $340 K$ (see Fig. S3 (a) and Fig. S3 (b)) which is comparable the experimental temperature rise of 10%. Our results indicate that the nanofluidic diode is thermally stable which helps for industrial scaling of the device. To the best of our knowledge these simulations are carried out for the first time in nanofluidic diode literature.


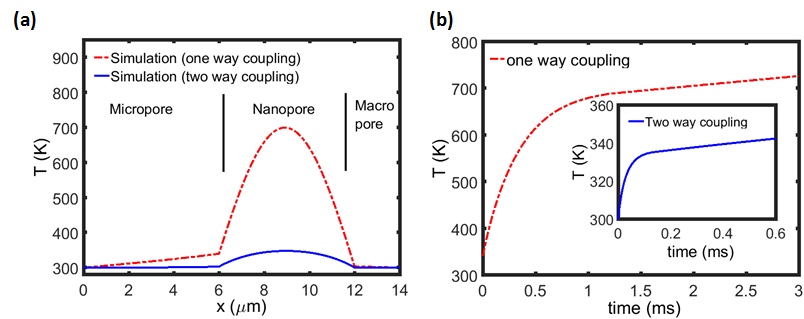


Figure S3 (a) Joule heating effect inside the nanofluidic diode using both one way and two ways coupling model (b) The variation of temperature with time inside the nanopore between the two models. We observe that the two ways coupling approach is more physical as it leads to 10% temperature rise when compared to one way coupling approach.

**Supplementary References:**

S1. V. V. R. Nandigana and N. R. Aluru, Nonlinear electrokinetic transport under combined ac and dc fields in micro/nanofluidic interface devices, *J. Fluids Engg*. **135**, 021201(2013).

S2. L. Verlet, Computer experiments on classical fluids. i. thermodynamical properties of Lennard-Jones molecules, Phys. Rev., **159**, 98–103(1967).

S3. W. H. Keesom, R. L. Zelenka and C. J. Radke, A Zeta-Potential Model for Ionic Surfactant Adsorption on an Ionogenic Hydrophobic Surface, *J. Coll. Int. Sci*., **125**, 575-585, 1988.

S4. B. Zaltzman and I. Rubinstein, Electro-osmotic slip and electroconvective instability, *J. Fluid Mech.* **579**, 173-226 (2007).

S5. I. Rubinstein and B. Zaltzman, Electro-convective versus electroosmotic instability in concentration polarization, Adv. Coll. Int. Sci., **134–135**, 190-200 (2007).

S6. S. Pennathur and J. G. Santiago, Electrokinetic Transport in Nanochannels. 1. Theory, *Anal. Chem*., **77**, 6772-6781 (2005).

S7. X. Xuan and D. Li. Analytical study of Joule heating effects on electrokinetic transportation in capillary electrophoresis. *J. of Chromat. A*, **1064**, 227–237 (2005).

S8. X. Xuan, D. Sinton and D. Li. Thermal end effects on electroosmotic flow in a capillary. *Int. J. of Heat and Mass Trans.***47**, 3145-3157 (2004).

S9. C. F. Beaton. Heat Exchanger Design Handbook (1986).

**Supporting information movie:** OFF state concentration polarization depletion and accumulation regions in the micro and macropore, respectively.


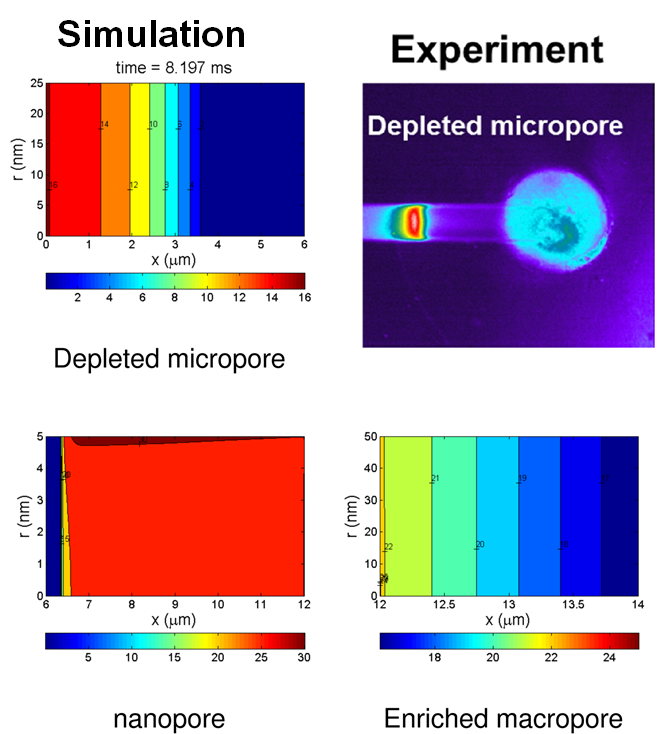

Supplement: Supplementary file 1 — Supplementary information [file 41598_2018_32284_MOESM1_ESM.docx]
